# Supplementary material for: Perceived social support and associated factors among older people living in metropolitan cities of northwest Ethiopia: A community-based cross-sectional study
Source: PLoS One. 2024 Nov 25;19(11):e0313996. doi: 10.1371/journal.pone.0313996 (PMC11588281; doi:10.1371/journal.pone.0313996)
Supplement: Attachment — Submitted filename: Reviewer comment.docx [file pone.0313996.s002.docx]

I applaud the author's motivation for putting this notion into action. Based on my rigorous review, I have some suggestions for improving the manuscript's quality and increasing its chances of approval. By expressing this, I intend to use both general and specific comments components appropriately.

**General Comments**

1. The article lacks continuous line numbers; why not put in line numbers? It is simple to provide comments and suggestions using point line numbers, and when addressing the offered remarks, remember to designate addressed comments with line numbers and page numbers.

2. What concerns me the most about this article are the tools used by the authors to assess the outcome variable perceived social support, and why you (authors) prefer FSSQ over more comprehensive tools used to assess perceived social support, such as the Multidimensional Scale of Perceived Social Support (MSPSS). FSSQ is typically used to assess the role of social support on medication adherence among patients with mental disorders, rather than general population perceived social support. Even it contains two components: confidant support and affective support, the scoring technique is continuous rather than dichotomous. How did you classify your outcome variable as low or high? This means that methods of scoring with suitable citation are required.

3. Why are you interested in this topic? What is the expected value provided by this work, given that perceived social support is a well-studied topic around the world, even among particular populations such as the elderly? So, in the introduction part, convey the peculiarity of this title boldly.

4. I came across a paper titled "Satisfaction with life and associated factors among elderly people living in two cities in northwest Ethiopia: a community-based cross-sectional study" while reading past research on perceived social support in Ethiopia and around the world. This paper is practically identical to the current paper, including the authors, sample size, study location, and study population, so could you please clarify this issue?

**Specific Comments/component wise comments**

**Abstract**

- Please replace "the purpose of this study was to **ascertain**" with appropriate action verbs or describe how you would **ascertain** perceived social support among the study population in sentence four of the Background paragraph.

**Methods**

- In the multivariate analysis, factors having a P-value ≤ 0.05 were considered statistically significant. Are there variables with a P-value of 0.05 that are deemed statistically significant? Although the scientific term is "p-value less than or equal to 0.05," most scholars prefer "p-value less than 0.05." That means, are there marginally significant variables?

**Results**

- In sentence three, it says "likely to have a severe mental illness"??? It does not make sense, so change it to "participants having severe mental illness" or "an individual having severe mental illness."

**Conclusion and recommendation**

- Your conclusion and recommendations should be based on your findings, which indicates that modifiable variables such as mental illness and lack of social participation should be addressed by stakeholders or future studies. illness"

**Introduction**

- Change the word "background" to "introduction."
- After writing an overview or explanation of social support, please explain what it means for older persons. That means add overview of older person.
- “As a result, research on the extent of perceived social support and the factors that influence is important and timely for older adults, the community, legislators, implementers, and other relevant organizations that work with older adults.” Please rephrase this sentence to make it a sense.

**Methods and Materials**

- **Study design**, **period**, and **setting**.... Add periods between design and area or settings, and **notify the reader what guidelines were used to guide the construction of the methodological design**.

SAMPLE SIZE DETERMINATION AND SAMPLING TECHNIQUE

- **First,** why did you choose to use proportions from a pilot study instead of previous studies in the same country or socioeconomic setting?
- **Second,** on line three, it states "22 design effect 2". Kindly clarify it. Is Design Effect 2 or 22?
- **Third,** use a formula editor, such as MathType, to clarify sample size calculation procedures for readers.
- **Fourth,** you stated that study participants were recruited using systematic random sampling from registration lists. Could you please share or write the interval used to pick them?
- **Finally,** a schematic presentation of the sampling technique for assessing perceived social support between the two cities and sub-cities should be included**.**

**Operational Definition**

- The authors' choice of this instrument over the Multidimensional Scale of Perceived Social Support, which has superior psychometric qualities, remains unclear. I require compelling justification.
- Operationalize what is “older people”, “Young – old”, “Middle – old”, and “Old-old” mean and use appropriate references.
- Substance addiction is listed as an explanatory variable, which requires operationalization using appropriate tools. Define the terms "ever substance users" and "current substance users." Refer to ASSIST tools.

**Results**

- Of the 830 participants, 816 were enrolled in the study. Explain why 14 were excluded. If there is missing ,explain way of management.
- What is your benchmark for categorizing variables such as money, living conditions, and family size?
- Table 1 has contradictory data, such as "Having children /live/…. Yes 764 (93.6)" and "Number of live children (n =766)".Please clarify whether this is correct or incorrect.

PREVALENCE OF PERCEIVED SOCIAL SUPPORT

- Use the word prevalence instead of magnitude
- When reporting the prevalence of perceived social support among older individuals, it is preferable to provide simply the prevalence (percentage) of low perceived social support with a 95% confidence interval because the prevalence of high perceived social support among these populations is already known.Because your stated low PSS.

HEALTH CONDITION OF THE STUDY PARTICIPANTS

- Please explain whether the chronic medical and mental diseases listed in Table 2 were diagnosed by a physician, as well as what specific illnesses included in the chronic medical illness category.

NUTRITIONAL, BEHAVIORAL, AND PSYCHOSOCIAL CHARACTERISTICS OF THE STUDY PARTICIPANTS

- As I have already gave you suggestion first define ever and currents substance use amend accordable, for example in Table 3 it says “Alcohol consumption within the past 12 months” ,there is no such assessment in the case of substance use, according to alcohol, smoking and substance involvement screening tool (ASSIST) tools using at least one of a specific substance (Alcohol, Khat, Cigarette, and other substances) for nonmedical purposes within last 3 months according to is regarded as **current substance users** and using at least one of any specific substance (Alcohol, Khat, Cigarette, and other substances) for nonmedical purposes at least once in a lifetime according to ASSIST refers **ever substance users** ,so correct according to this tools.

FACTORS ASSOCIATED WITH PERCIEVED SOCIAL SUPPORT OF ELDERLY PEOPLE

- Is your outcome variable life satisfaction or perceived social support???
- under the table 4 model fitness value should be added as footnotes.

**Discussion**

- As I previously stated, it is preferable to focus on low perceived social support, and a 95% confidence interval is essential when comparing your findings to research conducted around the world.
- In the second paragraph, is your study higher or lower than those of Spain and India? As far as I can see, your prevalence of low perceived social support is higher than studies conducted in both nations. So, please check and clarify for the reader. Include reasons for any discrepancies or differences in prevalence between your study and theirs. This includes the following: "In India 8.04%, 38.47%, and 53.48% of the elderly were found to have low, moderate, and high social support, respectively" …This category is based on the Oslo Social Support -3 items.
- Regarding factors associated with low perceived social support,you merely interpreted the findings; please include comparisons with other studies conducted around the world.

**Limitations**

- Add limitations to the study design as well.
- Furthermore, because it is self-reported data and a face-to-face interviewer-administered technique, there is the possibility of social desirability bias and amnesia, so consider these limitations as well.

**Conclusion and recommendations**

- It should be based on your findings, with a particular emphasis on modifiable factors identified as being connected with your outcome variable.

**That’s it for now, and I am looking forward for reading your manuscript in its final form.**
